# Exploring the Influence of Nanocrystalline Structure and Aluminum Content on High-Temperature Oxidation Behavior of Fe-Cr-Al Alloys

**DOI:** 10.3390/ma17071700

**Published:** 2024-04-08

**Authors:** Rajiv Kumar, R. K. Singh Raman, S. R. Bakshi, V. S. Raja, S. Parida

**Affiliations:** 1 Department of Metallurgical and Materials Engineering, Indian Institute of Technology Ropar, Bara Phool 140001, India; 2 IITB-Monash Research Academy, Indian Institute of Technology Bombay, Mumbai 400076, India; 3 Department of Metallurgical Engineering and Materials Science, Indian Institute of Technology Bombay, Mumbai 400076, India; vsraja@iitb.ac.in (V.S.R.); paridasm@iitb.ac.in (S.P.); 4 Department of Mechanical and Aerospace Engineering, Monash University, Clayton, VIC 3800, Australia; 5 Department of Chemical and Biological Engineering, Monash University, Clayton, VIC 3800, Australia; 6 Department of Metallurgical and Materials Engineering, Indian Institute of Technology Madras, Chennai 600036, India; sbakshi@iitm.ac.in

**Keywords:** nanocrystalline alloys, Fe-Cr-Al alloys, oxidation kinetics, oxidation resistance

## Abstract

The present study examines the high-temperature (500–800 °C) oxidation behavior of Fe-10Cr-(3,5) Al alloys and studies the effect of nanocrystalline structure and Al content on their resistance to oxidation. The nanocrystalline (NC) alloy powder was synthesized via planetary ball milling. The prepared NC alloy powder was consolidated using spark plasma sintering to form NC alloys. Subsequently, an annealing of the NC alloys was performed to transform them into microcrystalline (MC) alloys. It was observed that the NC alloys exhibit superior resistance to oxidation compared to their MC counterparts at high temperatures. The superior resistance to oxidation of the NC alloys is attributed to their considerably finer grain size, which enhances the diffusion of those elements to the metal–oxide interface that forms the protective oxide layer. Conversely, the coarser grain size in MC alloys limits the diffusion of the oxide-forming components. Furthermore, the Fe-10Cr-5Al alloy showed greater resistance to oxidation than the Fe-10Cr-3Al alloy.

## 1. Introduction

The materials used in high-temperature applications must exhibit the required resistance to oxidation at such temperatures. Fe-Cr-Al alloys are extensively used in high-temperature applications such as boilers/steam generators and as heating elements [1] for various applications such as furnaces, gas burners, furnace rollers, and ignitors. These materials are also commonly used in solar power systems as construction materials [2], automobiles as catalyst support [2], and nuclear power plants as fuel cladding materials against fuel accidents [2,3,4] because of their prominent resistance to high-temperature oxidation and neutron irradiation. Researchers have also investigated the oxidation characteristics of various Fe-Cr-Al alloys to identify conditions under which a protective α-Al_2_O_3_ layer can fully develop at high temperatures [1,2,5,6,7,8,9,10,11,12]. As α-Al_2_O_3_ is a much more defect-free oxide (than Cr_2_O_3_), an alumina layer provides superior protection at temperatures as high as 1350 °C [6,13]. During the oxidation of alumina-forming Fe alloys such as Fe-Al and Fe-Cr-Al alloys, less protective transients of Al_2_O_3_ (such as γ, θ, and δ-Al_2_O_3_) can form at temperatures below 900 °C, but it converts into the most stable form of Al_2_O_3_ (i.e., α-Al_2_O_3_) at higher temperatures. The formation of a robust layer of Al_2_O_3_ requires ~10–15 at% Al in Fe-Al alloys [14]. However, such high Al contents have a deleterious influence on the mechanical properties (particularly ductility), which restricts the use of such alloys in load-bearing structural components [11,15]. In this respect, it is highly attractive to find means for fully developing an alumina layer on Fe alloys, without using excessively high Al contents. One such approach is to use Fe-Cr-Al alloys, where the addition of Cr enables the development of an alumina layer at much lower Al contents. The chromium addition to Fe-Al alloys for remarkably lowering the critical content of aluminum required for the development of Al_2_O_3_ is known as the “Third Element Effect” of Cr [9,16,17], where the addition of chromium to Fe-Al alloys enables the full development of a protective layer of α-Al_2_O_3_ [5,9,17].

Studies [18] have established that a grain size reduction in Fe-Cr alloys to a nano-size regime reduces the critical concentration of oxide-forming element required for the development of a protective oxide layer. Similarly, investigations of the oxidation characteristics of Fe-Cr-Al alloys have also demonstrated that a reduction in grain size to a nano-regime reduces the critical concentration of aluminum required for the development of a robust Al_2_O_3_ layer [19,20]. The grain size reduction to nano levels enhances the diffusion through the grain boundary in the alloy by three to five orders of magnitude, which enables development of the protective oxide layer, thereby enhancing resistance to oxidation [21,22]. Another aspect is the spallation characteristics since Fe-Cr-Al alloys suffer oxide scale spallation under cyclic oxidation. In addition, Nanocrystalline (NC) structure can help resist oxide scale spallation (since the grain boundaries act as sites for oxide anchoring), which is beneficial for accommodating thermal stresses in the oxide scale, thereby resisting spallation, such as during thermal cycling [23,24]. These beneficial effects of nanocrystalline structure motivated us to investigate the influence of a nanocrystalline structure on the oxidation of Fe-Cr-Al alloys with different Al contents over a temperature range of 500–800 °C.

Although the beneficial effects of a nanocrystalline structure on oxidation are widely recognized, this aspect has been minimally explored for Fe-Cr-Al alloys, as most studies on the oxidation of Fe-Cr-Al alloys [1,5,9,10,16,25,26,27,28] have focused on microcrystalline (MC) alloys. However, we recently reported the influence of a nanocrystalline structure on Fe-Cr-Al alloys at various temperatures [19,20]. The oxidation behaviors of a Fe-20Cr-3Al alloy [20] and Fe-20Cr-5Al alloy [19] showed chromium to promote the formation of Al_2_O_3_. However, the combined influence of aluminum content and the nanocrystalline structure on the oxidation of Fe-Cr-Al alloys remains a relatively unexplored territory. Therefore, the present study primarily focused on examining the effects of aluminum content and a nanocrystalline structure on the oxidation behaviors of Fe-10Cr-(3,5) Al (wt%) alloys at 500, 700, and 800 °C. The resulting oxide scales were characterized using various characterization techniques to gain insights into their composition and structure.

## 2. Experimental Procedure

### 2.1. Synthesis of Nanocrystalline (NC) and Microcrystalline (MC) Fe-10Cr-(3,5) Al Alloys

Fe-10Cr-(3,5)Al alloys (NC and MC) were synthesized through a powder metallurgy route. Fe, Cr, and Al powders were ball milled to synthesize the NC Fe-10Cr-(3,5)Al alloy powders, following the reported procedure for the ball milling of Fe-10Cr-3Al alloys [29]. The consolidation of the milled Fe-10Cr-(3,5)Al alloy powder into a pellet with a 20 mm diameter was performed using spark plasma sintering (Dr. Sinter SPS-5000 Machine, Sumitomo Metals, Tokyo, Japan). The consolidation process utilized specifically optimized parameters: a temperature of 900 °C, pressure of 90 MPa, heating rate of 100 °C/min, holding time of 2 min, and a vacuum level of 0.1 Pa. The consolidated pellets are called NC Fe-10Cr-(3,5)Al alloys in the present study. Subsequently, the NC pellets were annealed in a tubular furnace at 900 °C for 20 h under a forming gas composed of 95% argon and 5% hydrogen. This annealing process transformed the NC structure into an MC structure, and the latter is called an MC Fe-10Cr-(3,5)Al alloy in the present study.

For an oxidation test, the surfaces of the consolidated alloy discs were polished using SiC papers up to a grit size of 2000, followed by a final polishing with a 0.1 µm diamond paste before oxidation. Isothermal oxidations of both the NC and MC Fe-10Cr-(3,5)Al alloys were conducted at 500, 700, and 800 °C in a tubular furnace for 60 h. The weights of the samples after oxidation at predetermined intervals of time were measured using an electronic balance (Sartorius CP225D, Göttingen, Germany). Subsequently, the oxidation kinetic plots were generated based on the collected weight gain data. The oxidation test runs were performed in triplicate for each test condition in order to examine the reproducibility.

### 2.2. Characterization of NC and MC Pellets before and after Oxidation

X-ray diffraction (XRD) patterns of the spark plasma sintered (SPSed) and annealed pellets of Fe-10Cr-(3,5) Al alloys were generated using a diffractometer (X’Pert pro, PANalytical, Almelo, The Netherlands) with CuK_α_ radiation (λ = 0.154056 nm), utilizing a step size of 0.02° and a duration of 20 s per step. The crystallite sizes of the alloys were calculated using the modified Williamson–Hall (MWH) technique [30] after considering peak broadening due to the XRD instrument, following a procedure described elsewhere [31].

The oxide scales formed upon oxidation at high temperatures were investigated using XRD. Field emission gun–scanning electron microscopy, FEG-SEM, (JEOL JSM-7600F, Tokyo, Japan) was used to characterize the surface morphology of the oxide layer. Elemental depth profiles of Fe, Cr and Al in the oxide scales developed in 60 h at different oxidation temperatures on both the NC and MC variants of the alloy were generated using time-of-flight secondary ion mass spectroscopy, TOF-SIMS (Physical Electronics/PHI TRIFT V NANO TOF, Chanhassen, MN, USA). TOF-SIMS depth profiles were obtained using a Cs^+^ ion primary sputter beam (energy: 2 keV, raster size: 50 μm, sputter time: 2 s), Ga ion analysis beam (energy: 30 keV, raster size: 800 μm), and current of 1.7 mA.

## 3. Results

### 3.1. Oxidation Kinetics

**Figure 1:**
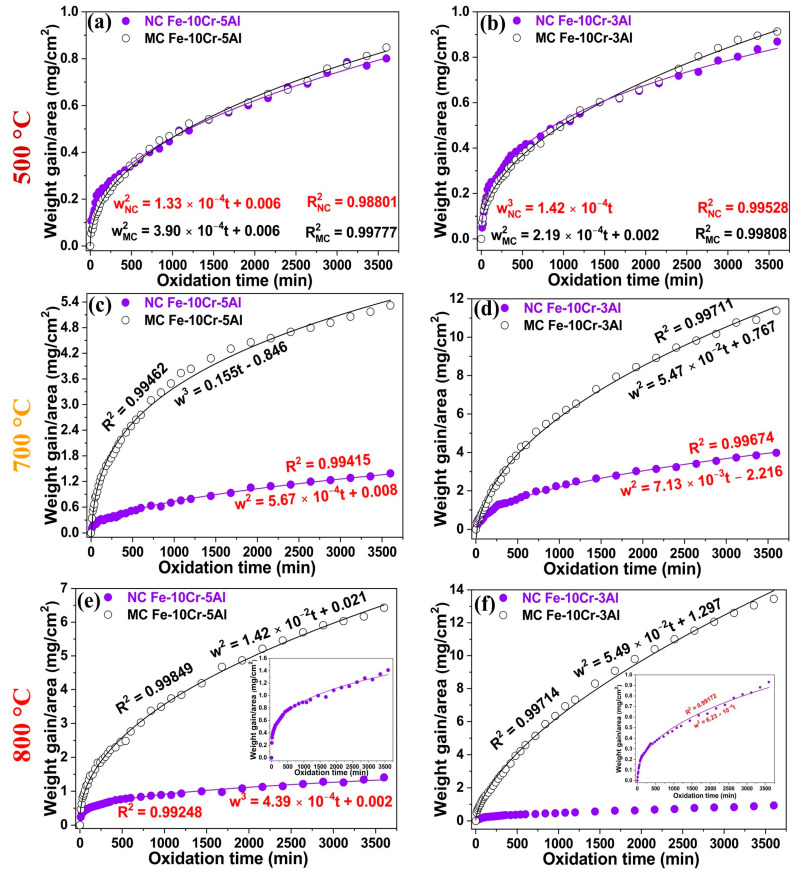
Oxidation kinetic plots for the Fe-10Cr-5Al alloy oxidized at (**a**) 500 °C, (**c**) 700 °C, and (**e**) 800 °C (inset, NC alloy) for 60 h. Oxidation kinetic plots for the Fe-10Cr-3Al alloy oxidized at (**b**) 500 °C, (**d**) 700 °C, and (**f**) 800 °C (inset, NC alloy) for 60 h.

The grain sizes of NC Fe-10Cr-3Al, NC Fe-10Cr-5Al, MC Fe-10Cr-3Al, MC Fe-10Cr-5Al were determined to be 91 ± 6 nm, 92 ± 8 nm, 0.9 ± 0.05 µm, and 0.7 ± 0.04 µm, respectively, following the procedure described in Section 2.2. The oxidation kinetics for the Fe-10Cr-5Al (Figure 1a) and Fe-10Cr-3Al (Figure 1b) alloys showed similar weight gains (w) after 60 h of oxidation at 500 °C, indicating that the NC structure has no discernible impact on the oxidation. However, the NC structure considerably influences the oxidation resistances of both the Fe-10Cr-5Al and Fe-10Cr-3Al alloys at 700 °C and 800 °C. The MC Fe-10Cr-5Al alloy exhibited about four and five times greater weight gains compared to the NC Fe-10Cr-5Al alloy after 60 h of oxidation at 700 °C (Figure 1c) and 800 °C (Figure 1e), respectively. Similarly, the MC Fe-10Cr-3Al alloy showed ~3 and ~14 times higher weight gains than the NC Fe-10Cr-3Al alloy after 60 h of oxidation at 700 °C (Figure 1d) and 800 °C (Figure 1f), respectively. The remarkable effect of a nanocrystalline structure on the oxidation resistance of the Fe-10Cr-5Al and Fe-10Cr-3Al alloys at the higher temperatures was investigated through a post-oxidation examination of the oxide scales developed on these alloys at 700 °C and 800 °C.

**Figure 2:**
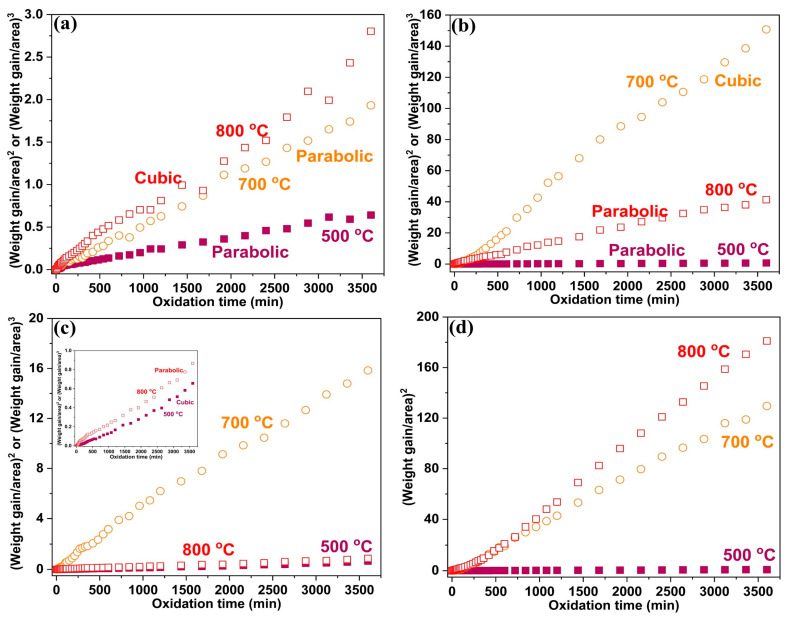
Oxidation kinetic plot for (**a**) NC Fe-10Cr-5Al, (**b**) MC Fe-10Cr-5Al, (**c**) NC Fe-10Cr-3Al, and (**d**) MC Fe-10Cr-3Al alloys oxidized at 500, 700, and 800 °C. The unit of weight gain/area is mg/cm^2^. If the (weight gain/cm^2^)^2^ verse oxidation time curve is linear, then the alloys follow parabolic laws; the alloys follow cubic laws if the (weight gain/cm^2^)^3^ verse oxidation time curve is linear.

The oxidation rate (rate of weight gain) generally increases with an increase in the oxidation temperature. Consistently, the Fe-10Cr-5Al and Fe-10Cr-3Al alloys showed notably higher weight gains at 700 °C than at 500 °C (Figure 2). However, upon a further increase in temperature to 800 °C, the NC Fe-10Cr-5Al alloy exhibited only a slight increase in weight gain relative to that observed at 700 °C after 60 h. On the other hand, the MC Fe-10Cr-5Al alloy showed a slightly higher weight gain at 800 °C after 60 h of oxidation. In the case of the Fe-10Cr-3Al alloy, the NC alloy exhibited a considerably lower weight gain at 800 °C compared to that at 700 °C, whereas the MC alloy exhibited a slightly higher weight gain at 800 °C compared to 700 °C. The increase in temperature (from 700 to 800 °C) has a relatively insignificant role in the oxidation resistances of the Fe-10Cr-5Al (NC and MC) and Fe-10Cr-3Al (MC) alloys, and their oxidation resistances are similar to that of the MC Fe-20Cr-3Al alloy at those temperatures [20]. This observation suggests that a more protective oxide scale forms on the Fe-10Cr-5Al and Fe-10Cr-3Al alloys at 800 °C compared to that at 700 °C. The oxidation kinetic constants (K_p_ for parabolic law and K_c_ for cubic law) for the Fe-10Cr-5Al and Fe-10Cr-3Al alloys were calculated from their weight gain plots (Figure 2) at 500, 700, and 800 °C, and they are listed in Table 1.

### 3.2. Oxide Morphology

**Figure 3:**
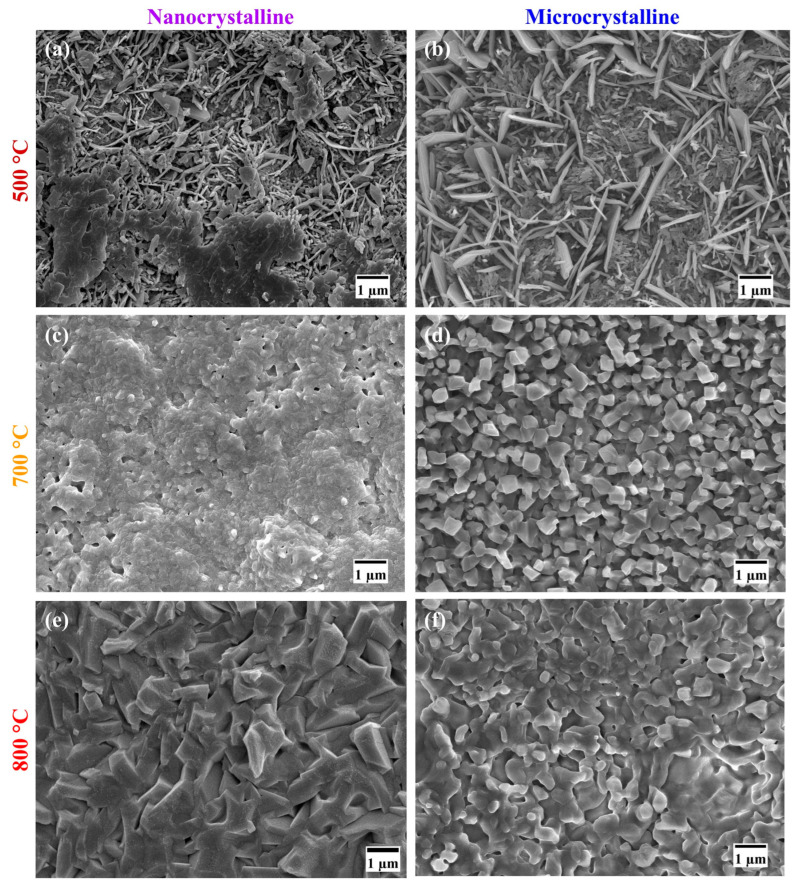
Morphologies of the oxide scales formed on the Fe-10Cr-5Al alloy in 60 h of the oxidation of the NC alloy at (**a**) 500 °C, (**c**) 700 °C, and (**e**) 800 °C and MC alloy at (**b**) 500 °C, (**d**) 700 °C, and (**f**) 800 °C.

**Figure 4:**
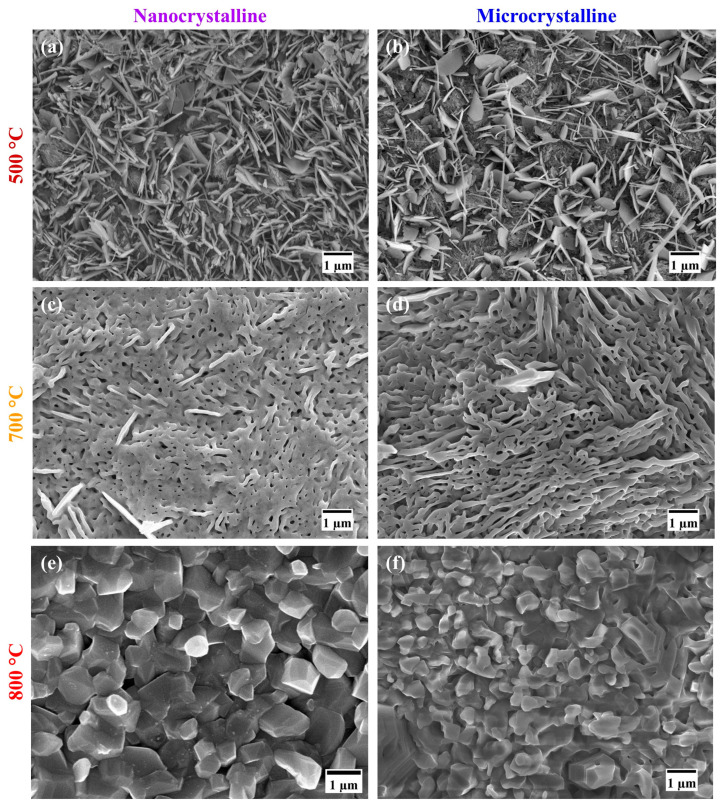
Morphologies of the oxide scales formed on the Fe-10Cr-3Al alloy in 60 h of the oxidation of the NC alloy at (**a**) 500 °C, (**c**) 700 °C, and (**e**) 800 °C and MC alloy at (**b**) 500 °C, (**d**) 700 °C, and (**f**) 800 °C.

The oxide scales developed on both the Fe-10Cr-5Al (Figure 3a,b) and Fe-10Cr-3Al (Figure 4a,b) alloys at 500 °C for 60 h exhibited similar oxide whisker/flake to the Fe oxides that are scattered over the entire surface. These flakes make the surface of the oxide scales porous and considerably reduce their protective properties. The broad similarity of the oxide scale morphologies of the Fe-10Cr-5Al and Fe-10Cr-3Al alloys consisting primarily of whiskers (Figure 3a,b) is consistent with the similar weight gains exhibited by the two after oxidation at 500 °C for 60 h (Figure 1a,b). However, the scale developed on the NC Fe-10Cr-5Al alloy at 700 °C appeared to be closely packed, faceted crystals of submicron size (Figure 3c), whereas a considerably porous, faceted crystal oxide formed on the MC Fe-10Cr-5Al alloy at 700 °C (Figure 3d). On the other hand, the MC Fe-10Cr-3Al alloy exhibited a porous oxide morphology at 700 °C, which was similar to that formed on its NC counterparts. In contrast, the oxide scales that developed on the Fe-10Cr-5Al (Figure 3e,f) and Fe-10Cr-3Al (Figure 4e,f) alloys at 800 °C were considerably compact than those formed at 700 °C. The oxide layers developed on the NC Fe-Fe-10Cr-5Al and NC Fe-10Cr-3Al alloys were considerably more compact with faceted crystals than those formed on their MC counterparts, indicating a positive effect of the NC structure on the oxidation resistances of these alloys.

### 3.3. Phase Composition for Oxide Scale

**Figure 5:**
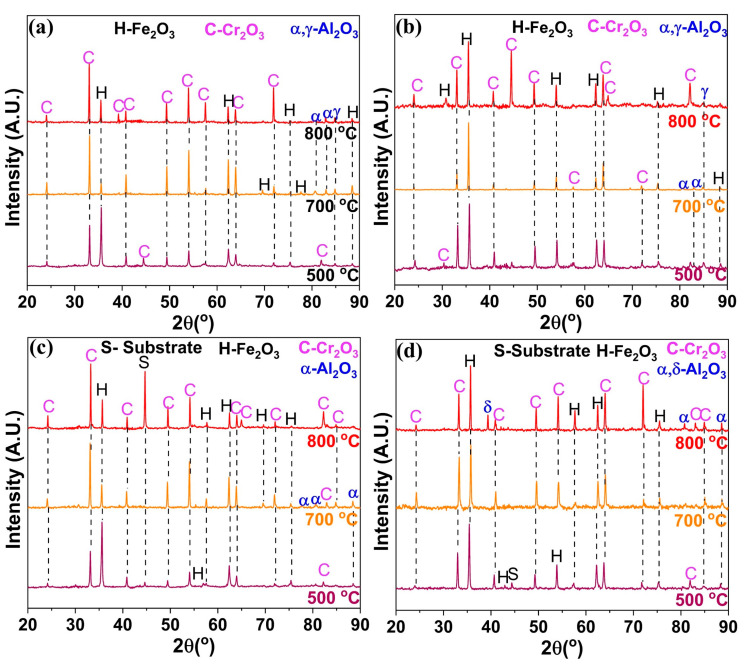
XRD profiles of oxide scales formed on (**a**) NC Fe-10Cr-5Al alloy, (**b**) MC Fe-10Cr-5Al alloy, (**c**) NC Fe-10Cr-3Al alloy and (**d**) MC Fe-10Cr-3Al alloy at 500, 700, and 800 °C in 60 h of oxidation.

Figure 5 shows the XRD spectra of oxides developed on the Fe-10Cr-5Al and Fe-10Cr-3Al alloys after 60 h at 500, 700, and 800 °C. The oxide scales developed on the Fe-10Cr-5Al and Fe-10Cr-3Al alloys after 60 h at 500 °C were predominantly composed of Fe_2_O_3_. In contrast, the oxide scales developed on the NC Fe-10Cr-(3,5)Al alloys at 700 and 800 °C after the same duration were rich in Cr_2_O_3_, while those on their MC counterpart at the same temperature after the same duration were rich in Fe_2_O_3._ The occurrence of Fe_2_O_3_-rich scales on the MC alloys is consistent with the higher weight gains exhibited by the MC alloys than those of the NC alloys at 700 and 800 °C (Figure 1).

### 3.4. TOF-SIMS Depth Profile of Oxide Scale

**Figure 6:**
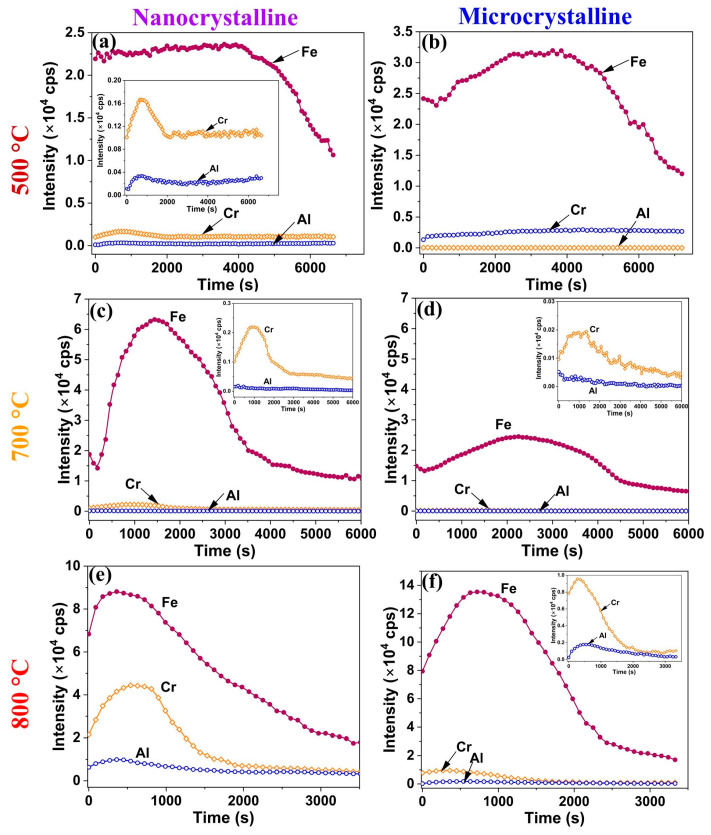
TOF-SIMS depth profiles of oxides formed on Fe-10Cr-5Al alloy in 60 h of oxidation of NC alloy at (**a**) 500 °C, (**c**) 700 °C, and (**e**) 800 °C and MC alloy at (**b**) 500 °C, (**d**) 700 °C, and (**f**) 800 °C.

**Figure 7:**
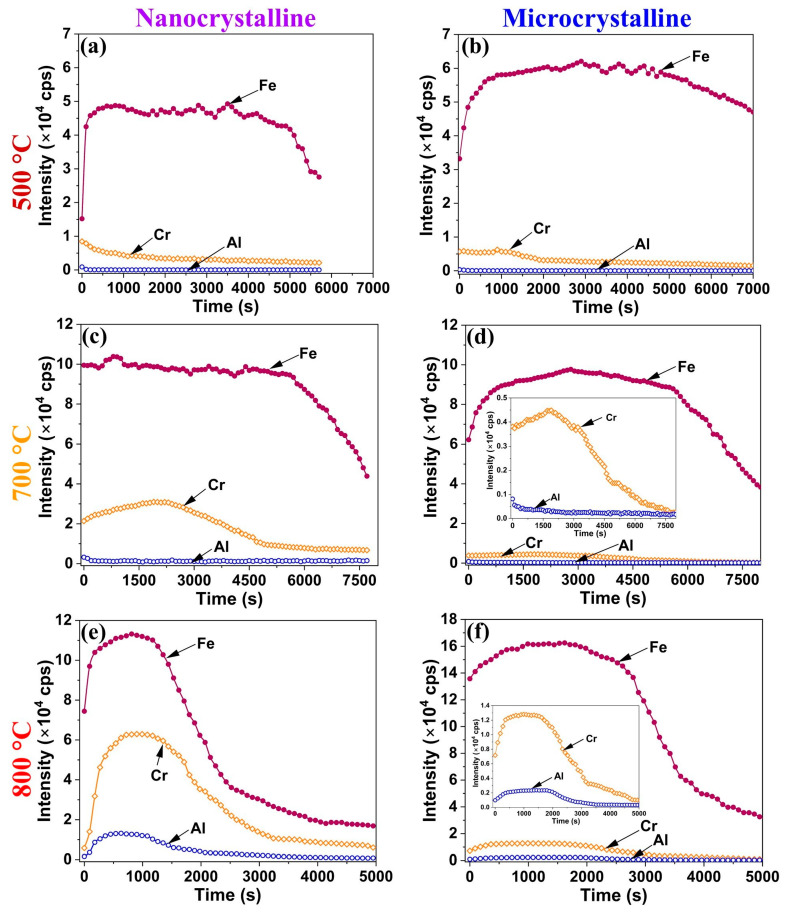
TOF-SIMS depth profiles of oxides formed on Fe-10Cr-3Al alloy in 60 h of oxidation of NC alloy at (**a**) 500 °C, (**c**) 700 °C, and (**e**) 800 °C and MC alloy at (**b**) 500 °C, (**d**) 700 °C, and (**f**) 800 °C.

TOF-SIMS profiles of the oxide scales developed on the Fe-10Cr-5Al (Figure 6a,b) and Fe-10Cr-3Al (Figure 7a,b) alloys after 60 h of oxidation at 500 °C are similar, indicating the formation of chemically similar (Fe_2_O_3_-rich) oxides on both the alloys. The broad Fe peak (TOF-SIMS profiles) indicates the development of a thick oxide on both of the Fe-10Cr-5Al and Fe-10Cr-3Al alloys at 500 °C. On the basis that the fast-growing iron oxide wustite (FeO) is thermodynamically stable only at temperatures above 570 °C, the broad Fe peak is attributed to the Fe_2_O_3_ that is stable at lower temperatures. However, the Fe_2_O_3_-rich oxide layer grows thick because the combined effect of diffusivity and the alloy microstructure is not able to facilitate a sufficiently rapid enrichment of Cr (or Al) that could facilitate the development of a contiguous layer of Cr (or Al) oxide. In this respect, it may be relevant to note that the oxidation of a Fe-10Cr alloy at temperatures such as 300 and 350 °C (at which the growth of the Fe_2_O_3_-rich oxide layer is sluggish) when combined with a nanocrystalline structure were found to facilitate sufficient Cr enrichment to enable the development of a contiguous layer of Cr oxide [18]. As a result, the NC Fe-10Cr alloy showed a remarkably superior oxidation resistance compared to its MC counterpart [18]. In contrast, as described earlier, the combination of 500 °C and an NC structure does not enable the development of a contiguous layer of Cr oxide in the cases of the Fe-10Cr-5Al and Fe-10Cr-3Al alloys, and hence, both the NC and MC alloys oxidized at similar rates at 500 °C (Figure 1a,b), which is duly corroborated by the similarity of the intensities and breadths of the TOF-SIMS profiles for Fe. However, the NC structure did facilitate greater Cr and Al accumulation in the oxide layer, as reflected in the minor Cr and Al peaks for the NC alloy in Figure 6a and Figure 7a.

The TOF-SIMS profiles for the oxide scales developed on the Fe-10Cr-5Al (Figure 6c,d) and Fe-10Cr-3Al (Figure 7c,d) alloys after 60 h of oxidation at 700 °C show the peaks of Fe along with Cr. The Fe peak for the MC Fe-10Cr-5Al alloy (at 700 °C) is considerably broader than that for the NC Fe-10Cr-5Al alloy. In addition, the intensities of the Fe and Cr peaks of the NC Fe-10Cr-5Al alloy are ~3 and ~10 times greater than those of the MC Fe-10Cr-5Al alloy. However, the intensity of the Cr peak in the case of the NC Fe-10Cr-3Al alloy is about six times higher than that of the MC Fe-10Cr-3Al alloy. These observations suggest that enhanced diffusivity in the NC Fe-10Cr-5Al and NC Fe-10Cr-5Al alloys facilitated the full development of an oxide on the NC alloys, aligning with their lower oxidation rate compared to the MC alloys at 700 °C.

The scales developed on the Fe-10Cr-5Al (Figure 6e,f) and Fe-10Cr-3Al (Figure 7e,f) alloys at 800 °C show low-intensity TOF-SIMS peaks of Fe, Cr, and Al. These peaks are considerably broader in the cases of the MC Fe-10Cr-5Al and MC Fe-10Cr-3Al alloys than their NC counterparts. Furthermore, the intensities of the Cr and Al peaks of the NC Fe-10Cr-5Al alloys surpass those of the MC Fe-10Cr-5Al alloys by ~5 and ~10 times, respectively, whereas the intensities of Cr and Al peaks of the NC Fe-10Cr-3Al alloys exceed those of the MC Fe-10Cr-3Al alloys by ~5 and ~6 times, respectively. The broad and low-intensity peaks of Cr and Al in the case of the MC alloys at 800 °C suggest a wider distribution of Cr and Al over a larger scale thickness, indicating a considerably less protective oxide formation on the MC alloys compared to the NC alloys. These observations are consistent with the lower weight gains of the NC Fe-10Cr-5Al and NC Fe-10Cr-3Al alloys compared to their MC counterparts at 800 °C (Figure 1e,f).

## 4. Discussion

Fe_2_O_3_-rich mixed oxide scales formed on the Fe-10Cr-5Al and Fe-10Cr-3Al alloys at 500 °C at the early periods of oxidation, which are similar to those formed on the Fe-20Cr-3Al alloy [20]. Upon further oxidation, the Fe_2_O_3_ scale grows rapidly in comparison to the Cr_2_O_3_ and Al_2_O_3_ due to an unavailability of sufficient Cr and Al for the formation of a protective oxide layer at the surfaces of the alloys. The formation of Fe_2_O_3_ also dominates over both the influences of Al addition and the impact of the NC structure on the alloys. Consequently, both the Fe-10Cr-5Al and Fe-10Cr-3Al alloys exhibit the formation of an Fe_2_O_3_-rich oxide and a similar oxidation behavior (parabolic oxidation kinetics) at 500 °C, indicating that the 10 wt% Cr is not sufficient for the development of a continuous layer of Cr_2_O_3_ at that temperature. However, the NC structure efficiently influences the oxidation behaviors of the Fe-10Cr-5Al and Fe-10Cr-3Al alloys at 700 °C. The NC structure of the alloys effectively enhances the diffusivities of the constituents of the alloys due to high grain boundaries. The higher diffusivities promote the formation of an oxide containing substantially more Cr_2_O_3_ in the NC alloy than that formed on the MC alloy (due to the limited diffusivity in the case of the latter). Consequently, the oxide scales developed on the NC Fe-10Cr-5Al and NC Fe-10Cr-3Al alloys consisted of a high proportion of Cr_2_O_3_ compared to their MC counterparts. The formation of a higher Cr_2_O_3_-containing oxide with faceted crystals in a scale morphology suggests the development of a more protective oxide layer on the NC alloys than on the MC alloys. Consequently, the NC Fe-10Cr-5Al alloy exhibits superior oxidation resistance than the MC Fe-10Cr-5Al alloy at 700 °C. Although the morphologies of the oxides developed on the NC Fe-10Cr-3Al and MC Fe-10Cr-3Al alloys at 700 °C are typically the same, the higher concentration of Cr_2_O_3_ in the oxide layer of the NC alloy enhances its resistance to oxidation compared to that of the MC alloy.

**Figure 8:**
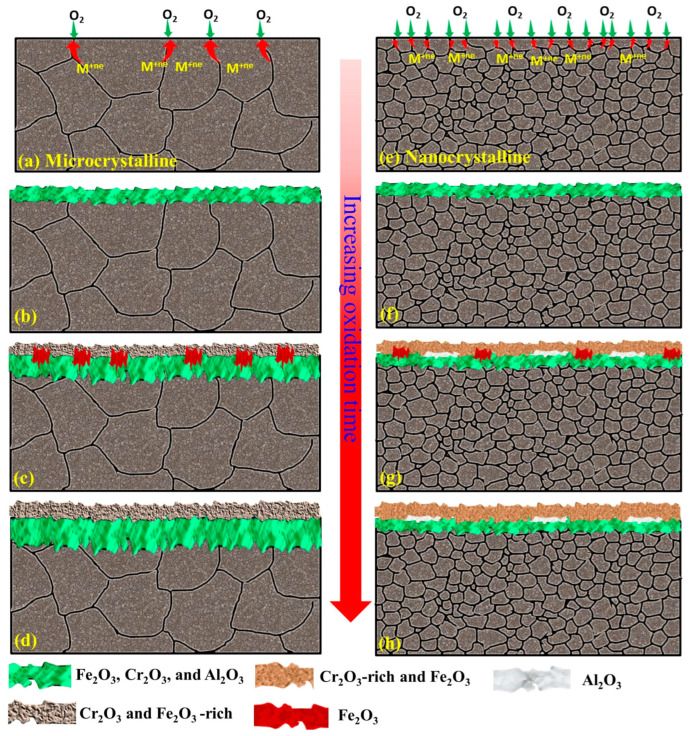
Schematic of plausible mechanism for oxide scale formation on microcrystalline (**a**–**d**) and nanocrystalline (**e**–**h**) Fe-10Cr-(3,5) Al alloys at high temperatures.

The impact of a nanocrystalline structure on the oxidation resistance behaviors of the Fe-10Cr-5Al and Fe-10Cr-3Al alloys at 800 °C is clearly exhibited by the oxide scales developed on these alloys. The compact and faceted crystals of the Cr_2_O_3_-rich oxide scales formed on the NC Fe-10Cr-5Al and NC Fe-10Cr-3Al alloys manifest in a noticeably lower weight gain than their MC counterparts. The oxide scales developed on the Fe-10Cr-5Al and Fe-10Cr-3Al alloys at the initial period of oxidation developed very rapidly at 800 °C compared those at 500 and 700 °C, especially for the NC alloy (Figure 8a,e). In addition, the diffusion coefficients of all constituents of the alloys at 800 °C are the same [32,33,34]. A rapid oxidation and similar diffusivity do not promote the formation of distinct oxide layers for all constituents of the alloys. As a result, the Fe_2_O_3_-rich mixed oxide scale forms at the initial period of oxidation (Figure 8b,f). Upon increasing the oxidation time, the oxide scale becomes enriched with Cr_2_O_3_, as Cr and Al have greater affinities for oxygen in comparison to Fe, and the Cr content in the alloys is higher than that of Al (Figure 8c,g). The formation of a continuous layer of a Cr_2_O_3_-rich oxide on the NC Fe-10Cr-5Al alloy impedes the diffusion of both metal and oxygen ions (Figure 8h), and hence, the NC Fe-10Cr-5Al alloy follows cubic oxidation kinetics. Conversely, the limited diffusion in the MC Fe-10Cr-5Al alloy restricts the development of a continuous layer of Cr_2_O_3_ on the MC alloy due to a lower diffusion coefficient of elements (Figure 8d). Hence, the MC Fe-10Cr-5Al alloy exhibited a higher oxidation rate compared to the NC Fe-10Cr-5Al alloy, and hence, the MC Fe-10Cr-5Al alloy follows parabolic oxidation kinetics. Further, the scale formed on the Fe-10Cr-3Al alloys follows a similar mechanism to that of the Fe-10Cr-5Al alloy. The post-oxidation characterizations of the oxides formed on the Fe-10Cr-5Al and Fe-10Cr-3Al alloys demonstrate the formation of an insignificant amount of Al_2_O_3_ on the alloys.

The characterization of an oxide scale after 60 h of oxidation using XRD and TOF-SIMS suggests that although there may be isolated instances of Al_2_O_3_ formation on the oxide scale, no continuous layer of protective Al_2_O_3_ formed on the oxide scale. Neither of the Fe-10Cr-3Al and Fe-10Cr-5Al alloys exhibited any formation of an Al_2_O_3_ layer after oxidation for 60 h at 500, 700 and 800 °C. The absence of a continuous Al_2_O_3_ layer indicates that the addition of 3 and 5 wt% Al with 10 wt% Cr in Fe is not sufficient for the full formation of a layer of Al_2_O_3_. However, our previous studies [19,20] suggest that 3 wt% Al and 5 wt% Al with 20 wt% Cr in Fe is sufficient for the full development of an Al_2_O_3_ layer.

## 5. Conclusions

The nanocrystalline (NC) structure of the Fe-10Cr-5Al and Fe-10Cr-3Al alloys does not enhance the oxidation resistance of the alloys at 500 °C. However, the NC structure plays a remarkable role in the oxidation resistances of the Fe-10Cr-5Al and Fe-10Cr-3Al alloys at higher temperatures. Fe_2_O_3_-rich oxide scales form at 500 °C on Fe-10Cr-5Al and Fe-10Cr-3Al alloys. In contrast, Cr_2_O_3_-rich scales form on NC Fe-10Cr-5Al and NC Fe-10Cr-3Al alloys at 700 and 800 °C, whereas Fe_2_O_3_-rich scales form on MC Fe-10Cr-5Al and MC Fe-10Cr-3Al alloys at these higher temperatures. A continuous layer of Al_2_O_3_ does not form on Fe-10Cr-5Al and Fe-10Cr-3Al alloys at the oxidation temperatures (500–800 °C), suggesting that added Al and Cr are not sufficient for the full formation of a layer of Al_2_O_3_.

## Figures and Tables

**Table 1:** Oxidation kinetic laws and kinetic constants of Fe-10Cr-5Al and Fe-10Cr-3Al alloys at 500, 700, and 800 °C.

Alloys	Temperature (°C)	Kinetic Law	Kinetic Constant
	500	Parabolic	K_p_ = 1.66 × 10^−10^ g^2^cm^−4^s^−1^
NC Fe-10Cr-5Al	700	Parabolic	K_p_ = 5.67 × 10^−10^ g^2^cm^−4^s^−1^
	800	Cubic	K_c_ = 4.39 × 10^−13^ g^3^cm^−6^s^−1^
	500	Parabolic	K_p_ = 3.90 × 10^−10^ g^2^cm^−4^s^−1^
MC Fe-10Cr-5Al	700	Cubic	K_c_ = 1.55 × 10^−14^ g^3^cm^−6^s^−1^
	800	Parabolic	K_p_ = 1.42 × 10^−8^ g^2^cm^−4^s^−1^
	500	Cubic	K_c_ = 1.42 × 10^−13^ g^3^cm^−6^s^−1^
NC Fe-10Cr-3Al	700	Parabolic	K_p_ = 7.13 × 10^−9^ g^2^cm^−4^s^−1^
	800	Parabolic	K_p_ = 6.23 × 10^−10^ g^2^cm^−4^s^−1^
	500	Parabolic	K_p_ = 2.19 × 10^−10^ g^2^cm^−4^s^−1^
MC Fe-10Cr-3Al	700	Parabolic	K_p_ = 5.47 × 10^−8^ g^2^cm^−4^s^−1^
	800	Parabolic	K_p_ = 5.49 × 10^−8^ g^2^cm^−4^s^−1^

## Data Availability

Data are contained within this article.
